# Routinely collected patient data in neurology research: a systematic mapping review

**DOI:** 10.1186/s12883-020-01993-w

**Published:** 2020-11-27

**Authors:** Fran Biggin, Hedley C. A. Emsley, Jo Knight

**Affiliations:** 1 Lancaster University Faculty of Health and Medicine, Furness College, Lancaster University, Bailrigg, Lancaster, LA1 4YG England; 2 Lancashire Hospitals NHS Foundation Trust, Department of Neurology, Royal Preston Hospital, Sharoe Green Lane, Fulwood, Preston, PR2 9HT England

**Keywords:** Neurology, Routinely collected data, Electronic health record

## Abstract

**Background:**

This review focuses on neurology research which uses routinely collected data. The number of such studies is growing alongside the expansion of data collection. We aim to gain a broad picture of the scope of how routine healthcare data have been utilised.

**Methods:**

This study follows a systematic mapping review approach which does not make a judgement on the quality of the papers included in the review, thereby enabling a complete overview of the field.

**Results:**

Of 4481 publications retrieved, 386 met the eligibility criteria for this study. These publications covered a wide range of conditions, but the majority were based on one or only a small number of neurological conditions. In particular, publications concerned with three discrete areas of neurological practice - multiple sclerosis (MS), epilepsy/seizure and Parkinson’s disease - accounted for 60% of the total. MS was the focus of the highest proportion of eligible studies (35%), yet in the recent Global Burden of Neurological Disease study it ranks only 14th out of 15 neurological disorders for DALY rates. In contrast, migraine is the neurological disorder with the highest ranking of DALYs globally (after stroke) and yet it was represented by only 4% of eligible studies.

**Conclusion:**

This review shows that there is a disproportionately large body of literature pertaining to relatively rare disorders, and a correspondingly small body of literature describing more common conditions. Therefore, there is potential for future research to redress this balance.

**Supplementary Information:**

The online version contains supplementary material available at 10.1186/s12883-020-01993-w.

## Background

The global burden of neurological disorders is increasing [1]. The Global Burden of Disease neurology collaborators reported that there has been a 39% increase in deaths due to neurological disorders between 1990 and 2016 [2]. Alongside this increase in the burden of disease, there is a predicted future shortfall in the US neurology workforce [3], and in the UK there is considerable concern surrounding services for people with neurological disorders [4–6]. A 2011 report by the UK National Audit Office (NAO) highlighted issues including delays in diagnosis, geographical inequalities in access to care; and a lack of good quality data [6].

Neurology is a large and diverse area of medicine with a correspondingly wide and varied body of research literature. Current neurology practice is heavily informed by the evidence provided by research, and the development of a focus on evidence based practice has been widely reported [7–9]. The use of data that have not been specifically collected for research is growing but we do not currently know how these data are being used in neurology research.

Routinely collected health data are collected from many different sources. For example, data may be collected at a patient’s face-to-face appointment with a healthcare professional, from administrative processes pertaining to the booking of the appointment, from laboratory results arising from tests requested at the appointment, for insurance information, or diagnostic coding for costing purposes [10]. Increasingly, health data are being recorded in an electronic manner, making it easier to store and access for research purposes.

Whilst the traditional hierarchy of evidence holds the randomised controlled trial (RCT) in highest regard, the use of routinely collected data to both supplement RCTs and conduct research outside of clinical trials is growing [8]. The 2018 scoping review for an extension to the Consolidated Standards of Reporting Trials (CONSORT) guidelines acknowledges the difficulties and limitations of RCTs and proposes that routinely collected data can be used to help address challenges such as cost, ‘limited real-world generalisability’ and recruiting representative samples to trials [11]. In addition, the use of routinely collected data to conduct stand-alone research is also being advocated. For example in their 2017 article Casey et al. explore in depth the advantages and disadvantages of using data obtained from the Electronic Health Record (EHR), a key source of routinely collected data, in population health research [12]. They conclude that research using EHRs has many advantages such as low cost, large sample sizes and the ability to link to other records, enabling, for example, the incorporation of social, behavioural and environmental data.

This review aims to explore how routinely collected patient data are currently being used in neurology research outside of clinical trials. We will take a broad view of the field in order to understand themes relating to study purpose, statistical methodology, and geographical location of the research. By understanding how routinely collected data are currently being used in neurology research this study intends to identify areas in which these data can be used to enhance future research.

## Methods

### Search strategy and selection criteria

Searches were carried out in eight online databases which span the topics of health, statistics, computing and general science. No restrictions were placed on the language of the research. All eight databases were searched between the 13th and 18th December 2018. No restriction was placed on the date of the publications to be retrieved; thus, the search was designed to retrieve all available studies published before December 2018. However, it is worth noting that the searches make use of the term Electronic Health Record (EHR) and as EHRs did not come into widespread use until the twenty-first century, the majority of studies retrieved were published after the year 2000. Searches were not restricted to full journal articles, allowing abstracts to be retrieved. Details of the search strategy and the databases searched can be found in the supplementary materials [Additional file 1].

In order to gain a large enough number of studies for analysis the searches were not limited by geographical location. However, this study concerned itself particularly with neurology research in the UK and so the search terms for the ‘neurology’ concept were developed using previous research carried out in the UK [13]. Specifically, neither stroke nor dementia were used as individual search terms as neither of these conditions are routinely seen in general neurology clinics in the UK, but rather, for the most part, in their own speciality settings [14].

### Data collection

Once the searches had been completed, 10 % of the retrieved papers were screened against draft eligibility criteria. This subset of the papers was then examined to refine the criteria. Following this initial screen the following eligibility criteria were defined.

Papers were included in this review if:

- Neurology or a neurological condition was the main focus of the study (excluding stroke and dementia).
- The study used only routinely collected data. This includes hospital records, primary care records, health insurance databases, and dispensary data.

Papers were excluded if:

- The primary focus was stroke or dementia.
- Any extra data were collected for the study. For example, patient questionnaires, focus groups or tests ordered specifically for the research.
- They were a systematic review or qualitative study.
- The population included individuals under 16 years of age.

These eligibility criteria were then applied to the whole set of retrieved papers. To reduce the impact of human error, 20% of the papers were audited by Emsley and Knight, ensuring consistent application of the criteria.

A data extraction form was used to extract relevant data from all eligible studies. See the supplementary materials [Additional file 1] for a table showing the data items extracted and used in the analysis.

The information required for the data extraction was taken from the study titles and abstracts. The full text of a paper was only retrieved if the necessary information could not be found in the abstract. Where possible, the variables were recorded verbatim as found in the paper. However, the information in the papers regarding study objective was not always explicitly clear so this was categorised whilst extracting the data. If the geographical location of the study was not explicitly mentioned in the paper then the country of the lead author’s first listed institution was taken as a proxy.

### Data analysis

Variables relating to neurological condition and statistical methodology were categorised, allowing for coherent analysis. The nine diagnosis categories used to analyse the data regarding the neurological condition(s) that formed the focus of the papers were defined using previous research and clinical expertise [13]. The statistical methodologies were categorised based on descriptive information contained within the individual articles combined with formal definitions of various statistical methodologies. Definitions for both the diagnosis and statistical categories can be found in the supplementary materials [Additional file 1].

## Results

**Fig. 1:**
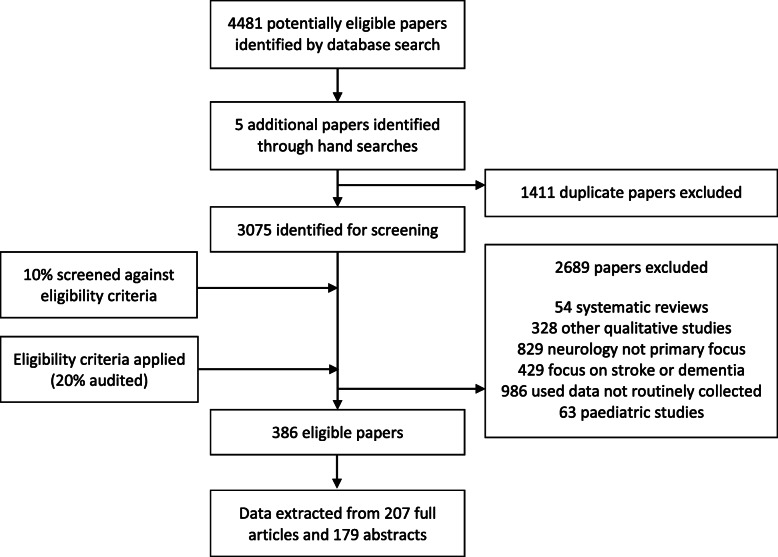
Flow chart showing study selection procedure

We retrieved 4481 papers from our database searches and five further papers by searching citations by hand. Once duplicates had been removed, 3075 papers remained for screening. The eligibility criteria were applied to these 3075 papers and 386 papers were deemed eligible for this study. Of these 386 papers, 207 were full research articles and 179 were abstracts only. This selection process can be seen in Fig. 1.

**Fig. 2:**
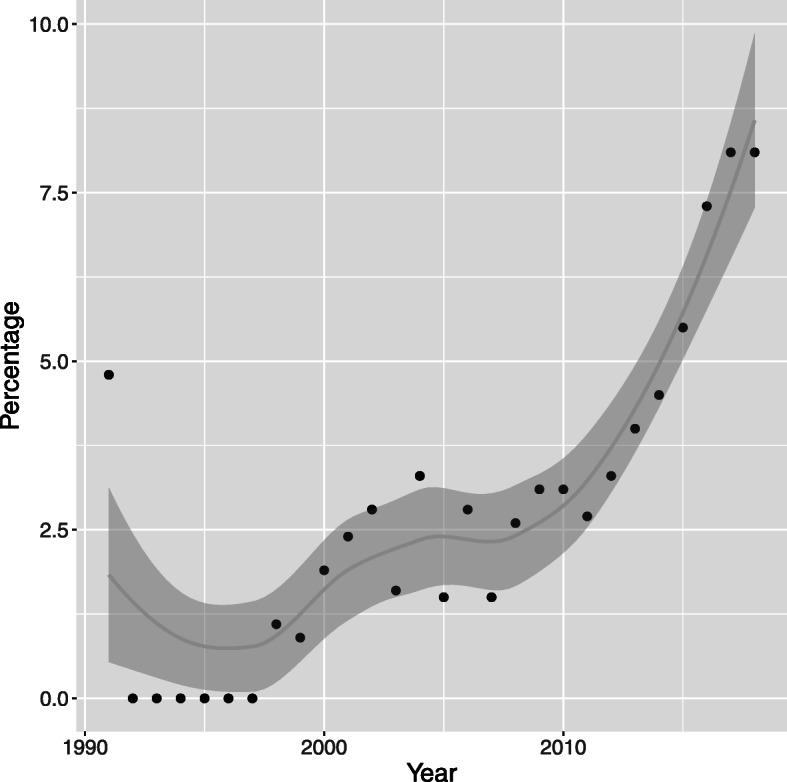
Neurology studies as a percentage of all medical studies. A graph of neurology papers as a percentage of all papers relating to the use of EHRs and Routinely Collected Data each year retrieved from a PubMed search

We compared the number of papers retrieved by our search in PubMed to an equivalent search on all medical papers. Overall, there are relatively few papers using EHRs and routinely collected data until around the year 2000, since when the number of papers has increased steadily. The earliest neurology specific paper was published in 1991, and the numbers follow the same general upward trend (see supplementary materials [Additional file 1] Table vi). Figure 2 shows that as a percentage of all medical papers referencing EHRs and routine data, neurology accounted for between 0 to 3.3% until 2012, apart from in 1991 when the single neurology paper published accounts for 4.8% of all papers. Since 2012 this percentage has been steadily increasing to reach 8.1% in 2017 and 2018.

An overview of the characteristics of the included papers can be seen in Table 1. They have been split into two separate columns – one for full articles and one abstracts only. This distinction has been made as many abstracts become, or contribute in a large part, to future full articles and it is not always possible to identify when this has occurred.

**Table 1:** Overview of study characteristics

Data Item	Category	Full Articles (*n* = 207, %)	Abstract Only (*n* = 179, %)
Neurological Condition	Multiple Conditions	4 (1·9)	7 (3·9)
	Single Conditions:		
	Multiple Sclerosis	61 (29·5)	78 (43·6)
	Epilepsy/Seizure	42 (20·3)	21 (11·7)
	Parkinson’s Disease	15 (7·2)	14 (7·8)
	Headache (all)	18 (8·7)	12 (6·7)
	Migraine only	10 (4·8)	6 (3·4)
	Neurodegenerative Disorders	6 (2·9)	2 (1·1)
	Neuromuscular Disorders	5 (2·4)	4 (2·2)
	Other	56 (27·1)	41 (22·9)
Statistical Methodology	Descriptive	127 (61·3)	116 (64·8)
	Regression	35 (16·9)	33 (18·4)
	Survival Analysis	12 (5·8)	8 (4·5)
	Administrative Data Algorithm	9 (4·3)	6 (3·3)
	Machine Learning	5 (2·4)	2 (1·1)
	NLP	5 (2·4)	5 (2·8)
	Propensity Scoring	4 (1·9)	4 (2·2)
	ANOVA	3 (1·4)	1 (0·6)
	Other	7 (3·4)	4 (2·2)
Study Objective	Characterisation of a clinical population	46 (22·2)	44 (24·6)
	Risk Factors	42 (20·3)	31 (17·3)
	Drug Effectiveness	26 (12·6)	15 (8·3)
	Prediction	18 (8·7)	13 (7·3)
	Healthcare Utilisation	13 (6·3)	9 (5·0)
	Diagnosis Validity	13 (6·3)	5 (2·8)
	Prevalence	9 (4·3)	7 (3·9)
	Drug Safety	9 (4·3)	5 (2·8)
	Drug Adherence	8 (3·9)	8 (4·5)
	Other	24 (11·6)	42 (23·5)
Data Type	Hospital Data	91 (44·0)	66 (36·9)
	Claims Data	22 (10·6)	44 (24·6)
	Clinic Data	30 (14·5)	28 (15·6)
	Multicentre Data	23 (11·1)	21 (11·7)
	Veterans or Military Data	13 (6·3)	11 (6·2)
	Primary Care Data	16 (7·7)	2 (1·1)
	Pharmaceutical Data	3 (1·5)	6 (3·4)
	Other	9 (4·3)	1 (0·6)
Location	USA	112 (54·1)	127 (70·9)
	Europe	54 (26·1)	30 (16·8)
	Rest of World	41 (19·8)	22 (12·3)

**Fig. 3:**
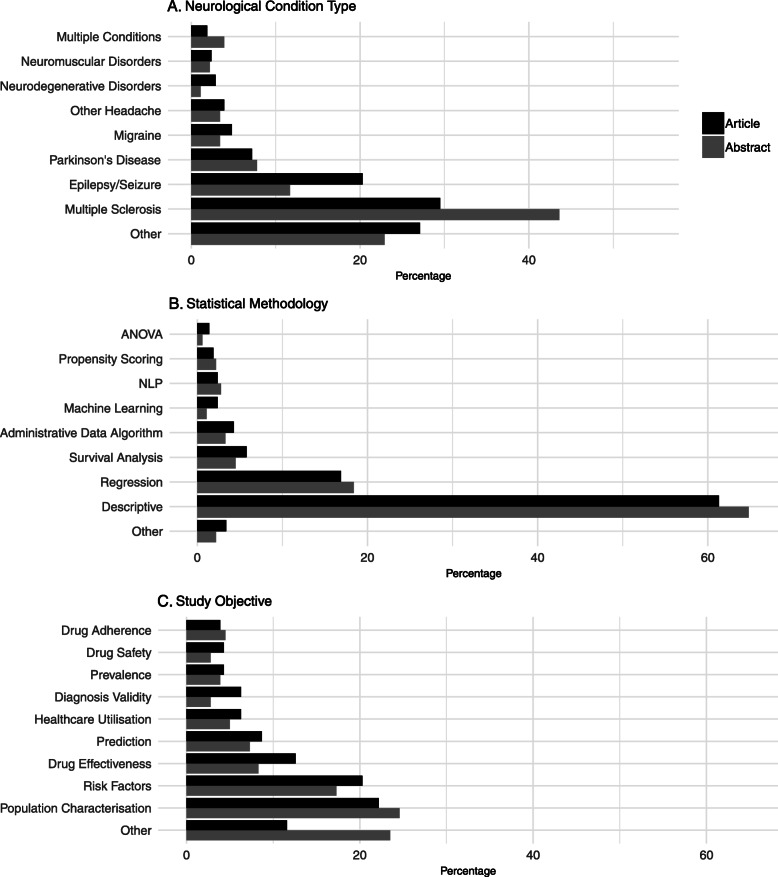
Visualisation of study characteristics. A breakdown of 3 of the variables extracted from each study. Panel **a** shows the percentage of studies focused on each neurological condition, **b** shows the percentage of studies using different statistical methodologies and **c** shows study objectives

Most of the papers, both full articles and abstracts, focus on a single type of neurological condition, with only four articles and seven abstracts referring to studies analysing data from multiple conditions. The most frequently studied condition in this analysis is multiple sclerosis (MS), followed by epilepsy/seizure and Parkinson’s disease (PD), which can be clearly seen in Fig. 3a. When comparing this to the global burden of neurological disorders we see that the frequency of the conditions studied does not reflect the burden of those conditions in the population [2]. Setting aside stroke and dementia (as they were specifically excluded from this study for reasons previously explained in the methods section) the top three neurological conditions ranked by age-standardised DALY (Disability-adjusted Life Years) rates in both Western Europe and North America are: migraine, spinal cord injury, and brain and central nervous system cancer.

In this review MS is the most frequently studied condition, yet globally it ranks only 14th out of 15 neurological disorders for DALY rates. In contrast, migraine is the neurological disorder with the highest ranking of DALYs globally (after stroke) and yet in this study we see only 4·8% of the full articles and 3·4% of the abstracts focus on this condition. This may reflect a number of issues such as the perception of the validity of research into a condition within the research community, the ease with which the condition can be studied, the availability of data and the availability of funding.

There are 11 papers which analyse many different neurological conditions together and are categorised as ‘multiple conditions’, four full articles and seven abstracts. Six of these papers cover a wide range of neurological conditions, however five of them focus on subsets of neurological conditions such as those treated with immunoglobulins [15, 16], neurologic emergencies [17, 18], and neuro-ophthalmology [19].

Figure 3b shows that the majority of the papers (61·3% of the full articles and 64·8% of the abstracts) exclusively used descriptive statistics in their analysis. This includes means, proportions and statistical tests such as t-tests used to test hypotheses on single variables. Of those papers that moved beyond descriptive statistics the most common type of statistical modelling used is regression modelling (16·9% of full articles and 18·4% of the abstracts). The benefit of using these forms of modelling over hypothesis testing on descriptive statistics is that the effect of many variables can be taken into account at once.

A small number of papers used methods which build on similar foundations to regression modelling including survival analysis (12 articles and eight abstracts) and propensity scoring (four articles and four abstracts). Other papers used a completely different approach to analysis using algorithmic methods. A small number of papers (nine articles and six abstracts) were dedicated to developing administrative data algorithms. Typical of these papers is Ho C et al. who used a set of rules applied to data stored in a discharge database to identify patients with non-traumatic spinal cord dysfunction [20].

There were relatively few papers using computationally intensive methods such as Natural Language Processing (NLP) (five articles and five abstracts) and machine learning (ML) (five articles and two abstracts). However, those few papers which have taken advantage of the large body of ‘Big Data’ available in routinely collected health records have used some innovative techniques. For example Chase et al. used NLP with a naïve bayes classifier to identify patients with MS from the EHR, demonstrating how analysing large amounts of routinely collected data could lead to early diagnosis of a neurological illness [21].

The majority of the studies used hospital data in their research (44% of full papers and 37% of abstracts), with the use of claims data second most common but more prevalent in abstracts (25%) than full papers (11%). Data from specialist clinics accounts for 14.5% of the full papers and 15.6% of abstracts. In addition, Tables vii and viii in the supplementary materials [Additional file 1] give a more detailed breakdown of the data type used for each condition (Table vii), and the types statistical analysis used for each data type (Table viii). From Table vii we see that for studies focusing on Multiple Sclerosis claims data was most commonly used (36% of studies), however conclusions are hard to draw regarding other conditions due to sample sizes. Table viii shows that descriptive analyses are the most common across all data types, however the distribution of statistical analyses used does vary across different data types.

The studies included in this review had a number of different study objectives, as can be seen in Fig. 3c. The most common objectives for both full articles and abstracts are ‘Characterisation of a clinical population’ and ‘Risk factors’. Characterising a clinical population refers to those types of study which seek to describe a group, or groups, of patients. For example in their 2016 paper Kestenbaum M et al. describe the characteristics of patients with either Parkinson’s disease or essential tremor who underwent deep brain stimulation [22]. In contrast, the studies regarding risk factors focus more on the factors leading to a disease or outcome, for example Modi SY et.al. published a paper examining the predictors of long hospital stays in status migrainosus [23].

Other common study objectives include research on drug effectiveness, safety and adherence. Taken together these types of study account for 20·8% of the full articles and 15·6% of the abstracts. The most common condition investigated by these types of study is MS, with two thirds of all the drug studies dedicated to this condition.

The vast majority of included studies were based in the USA (54·1% of full articles and 70·9% of the abstracts), 26·1% of the full articles were from Europe and 19·8% from the rest of the world. Of all the European papers eligible for inclusion in this study 29 were UK based, 14 of which were abstracts and 15 full articles. All of the UK based research focused on single types of neurological condition with epilepsy/seizure being the most commonly researched (seven papers), followed by Parkinson’s disease (four papers) and MS (four papers) showing a broadly similar trend to that seen in the whole body of eligible studies. However, we did not find a large enough number of UK based studies to do a full mapping review on this subset of research.

## Discussion

This study synthesises and summarises neurological research that has been carried out using routinely collected data, that is, data which were not initially collected for research purposes, but for reasons such as diagnosis, treatment or administration.

The results show that routinely collected patient data has been used for a number of different purposes in neurology research. Primarily, the data has been used to study single neurological conditions in isolation. Within these papers we found a variety of study objectives, the most common of which relate to the characterisation of a population, risk factors for an outcome and drug safety, adherence and effectiveness outside of clinical trials. Whilst these conditions are well researched, this study highlights the fact that there are potentially areas of neurology which remain under-researched in comparison.

There is an imbalance between the numbers of papers found for particular types of conditions, and the impact of those conditions (measured in DALYs) according to the global burden of neurological disease [3]. This indicates that there may be an opportunity for high impact research to take place into conditions that have a very real effect on healthcare systems, on society, and on individual patient’s lives. Previous research has highlighted the fact that there is an imbalance between the amount of research conducted and the rarity of a condition, with rare neurological conditions receiving disproportionately more attention than common ones [24, 25]. Bishop proposes that the reason that rare conditions receive more research focus is because of their severity [24], and Al-Shahi et al. propose that the amount of research conducted should be proportional to the burden of the disease in society [25]. A high rate of DALYs indicates the potential economic and social cost of common conditions such as migraine. Research into these less well-studied areas using routinely collected data could contribute to reducing the burden of disease and consequently the economic and social cost.

The statistical methodologies used in the papers included in this study range from descriptive statistics to more complex analyses based on Machine Learning techniques. Machine Learning techniques generally require large amounts of data from which to ‘learn’ a mathematical model which can then be applied to an unseen set of data to predict or classify future results. As the amount of routinely collected data grows, this is an area in which future neurology research could have an impact – for example by using Machine Learning to find previously unknown associations, or for phenotyping diseases [26]. However, the use of complex algorithms and computationally intensive methods relies on having the right kind of question as well as suitable data. This study shows that there are differences in statistical analyses used on different types of data (see table viii in Supplementary Materials [Additional file 1]). For example, the relatively high number of regression analyses undertaken on claims data may occur because claims data is often highly numerical and abundant, and therefore lends itself to this type of analysis. In addition, data from hospital records can be highly complex and include pages of written notes, and so we see that analyses using Machine Learning and NLP are used in these types of data. It is worth noting that not all types of data lend themselves to complex analyses, and statistical analyses should only be as complex as is required to answer the question at hand.

As expected, this review did not identify many studies using routinely collected data to investigate neurology services managing multiple conditions, such as outpatient clinics. Rather, this review clearly shows that the majority of research relates to single conditions or condition types such as epilepsy and MS. We found only 11 studies which included multiple conditions, and of those, only four were studies into the provision of services. Many neurology clinics provide treatment and care to patients with a wide range of conditions and as such, research relating to these services should incorporate all of those conditions [14]. There is a real opportunity here for research to be conducted using routinely collected data which could be used in many different ways to support the efficient delivery of services. For example, in other disciplines, routinely collected data have been used to examine waiting times for appointment and explore patient visit patterns [27, 28].

### Limitations

Systematic mapping reviews, like all systematic reviews have some underlying limitations, which include reporting and selection biases and inaccuracies in data extraction. Particular to mapping reviews is the issue of oversimplification – because a mapping review is designed to give a broad overview of an area, it can mask underlying variations in the included studies [29]. In this study we have sought to limit the impact of reporting bias (the tendency for research with positive studies more likely to be published) by searching for and including papers that have been published as abstracts. This ensures that research in emerging areas is included, as well as studies that have perhaps not yet merited full publication.

Selection bias was limited by defining strict eligibility criteria before the papers were screened for inclusion. The application of the eligibility criteria to the list of potential papers was also quality assured in 20% of the papers to ensure that the criteria were applied consistently.

Other limitations include inaccuracy in data extraction and classification, which is inevitable when using categories to define study characteristics, however we have been consistent throughout the study and the definitions used for the categories can be found in the supplementary materials.

Applying the results of this review across different geographical areas should be done with caution. The majority of the studies in this review were conducted in the USA and Western Europe where neurology services and policies may differ significantly from other areas with different healthcare structures and populations. Even within Western Europe there are many differences in the way in which services are delivered and the data recorded [30]. Future studies should endeavour to relate the findings of this review to their own context, and as more neurology research emerges in different countries and contexts, the gaps in research in individual areas will become clearer. In addition, applying conclusions drawn from the location of the studies should take into account the fact that study location was not always explicit. In these cases, study location was taken to be the location of the lead author’s main institution.

The main strength of this review is that research on neurological conditions using routinely collected data has not been reviewed in this way before. This study allows us to see what work is already being done, and where future research could have an impact. As with all systematic reviews the methodology of this study has been well documented such that it could be repeated and the results replicated in the future.

## Conclusion

There is a large body of research within neurology that exclusively uses routinely collected data, including data from electronic health records, public health records, and primary care data as well as administrative data such as medical insurance claims. This research covers a wide range of conditions, outcomes and study objectives. We have discovered an underrepresentation of studies into common conditions. It is also clear from this study that there are few studies which include multiple conditions in the same research, or which study neurology services as a whole. Future research using routinely collected data could make a large impact by considering the more common but less well-researched conditions or by considering how services could be improved by utilising data from many conditions.

## Supplementary Information

**Additional file 1:** Detailed Search Strategy; Data Items Extracted; Definitions; and Supplementary Results Tables.

## Data Availability

The datasets used and/or analysed during the current study are available from the corresponding author on reasonable request.

## Abbreviations

CONSORT: Consolidated Standards of Reporting Trials
DALY: Disability Adjusted Life Years
EHR: Electronic Health Record
ML: Machine Learning
MS: Multiple Sclerosis
NAO: National Audit Office
NLP: Natural Language Processing
PD: Parkinson’s Disease
