# Supplementary material for: Routinely collected patient data in neurology research: a systematic mapping review
Source: BMC Neurol. 2020 Nov 27;20:431. doi: 10.1186/s12883-020-01993-w (PMC7694309; doi:10.1186/s12883-020-01993-w)
Supplement: Supplementary file 1 — Additional file 1: Detailed Search Strategy; Data Items Extracted; Definitions; and Supplementary Results Tables. [file 12883_2020_1993_MOESM1_ESM.docx]

**Supplementary Materials**

**Search Strategy**

| 1 | electronic medical record [abstract/title] |
| --- | --- |
| 2 | electronic health record [abstract/title] |
| 3 | electronic patient record [abstract/title] |
| 4 | EHR [abstract/title] |
| 5 | real world data [abstract/title] |
| 6 | real world evidence [abstract/title] |
| 7 | routinely collected data [abstract/title] |
| 8 (PubMed only) | Electronic health records [Mesh] |
| 9 | 1 or 2 or 3 or 4 or 5 or 6 or 7 or 8 |
| 10 | (migraine or headache or headache disorders) [abstract/title] |
| 11 | (functional neurological disorder or functional neurological symptom disorder) [abstract/title] |
| 12 | (epilepsy or seizure) [abstract/title] |
| 13 | (multiple sclerosis or MS or demyelination) [abstract/title] |
| 14 | (degenerative spine disease or myelitis) [abstract/title] |
| 15 | (parkinson’s disease or parkinsons disease or tremor or dystonia or parkinsonism) [abstract/title] |
| 16 | (syncope or transient loss of consciousness) [abstract/title] |
| 17 | (peripheral nerve disorder or polyneuropathy or mononeuropathy) [abstract/title] |
| 18 | neurol* [abstract/title] |
| 19 (PubMed only) | Nervous Systems Disease [Mesh] |
| 20 | 10 or 11 or 12 or 13 or 14 or 15 or 16 or 17 or 18 or 19 |
| 21 | 9 and 20 |

Table i. Search strategy used for the systematic mapping review.

**Databases Searched**

| 1 | PubMed |
| --- | --- |
| 2 | EMBASE: Excerpta Medica |
| 3 | CINAHL |
| 4 | Academic Search Ultimate |
| 5 | SCOPUS |
| 6 | Web of Science |
| 7 | MathSciNet |
| 8 | IEEExplore |

Table ii. Databases searched for the systematic mapping review.

**Data Items Extracted**

| **Variable** | **Collected As** | **Categorised to** |
| --- | --- | --- |
| Neurological Condition | Free text | 9 diagnostic categories |
| Statistical Methodology | Free text | 10 methodology categories |
| Study Objective | Category |  |
| Data Type | Category |  |
| Study Location | Free text |  |

Table iii. All data items extracted from the eligible papers.

**Definitions**

| Data Item | Category | Description |
| --- | --- | --- |
| Statistical Methodology | Descriptive | Analysis limited to descriptive statistics such as means and proportions, and hypothesis tests on single variables. |
|  | Regression | Statistical modelling using regression models. Includes all forms of regression including both linear and logistic regression. |
|  | Administrative Data Algorithm | Deterministic algorithms applied to patient data. Generally used to identify patients with certain diagnoses. |
|  | Survival Analysis | Modelling designed to analyse survival times. Include Kaplan Meier and Cox proportional hazards. |
|  | ANOVA | Statistical modelling using Analysis of Variance. |
|  | Natural Language Processing | Analysis of large amounts of text using computer science and computational linguistics. |
|  | Propensity Scoring | The use of propensity score matching statistical matching technique |
|  | Machine Learning | Analysis using algorithms where the computer learns from the data. Generally used for prediction. |
|  | Other | Any other statistical or analytical technique not otherwise described here. |

Table iv. Definitions of the statistical methodology categories used in the analysis.

| Data Item | Category | Description |
| --- | --- | --- |
| Neurological Condition | Multiple conditions | Papers which analyse many different neurological conditions and diagnoses at once. For example, papers which consider all types of neurologic emergency. |
|  | Multiple Sclerosis | Papers examining all four types of Multiple Sclerosis. |
|  | Epilepsy/Seizure | Papers examining all types of epilepsy and seizure disorders. |
|  | Parkinson’s Disease | Papers examining Parkinson’s Disease, Parkinsonism and Essential tremor. |
|  | Headache (all) | Papers examining all types of primary headache including cluster headache, migraine and tension headache. |
|  | Migraine only | Those papers which exclusively examine migraine. |
|  | Neurodegenerative Disorders | Papers examining Neurodegenerative disorders other than Parkinson’s disease, which has its own category, and Alzheimer’s, as dementia papers were removed from the study. |
|  | Neuromuscular Disorders | Papers examining Neuromuscular disorders other than Multiple Sclerosis, which has its own category. |
|  | Other | Papers examining all other neurological conditions and diagnoses which do not fit into one of the above categories. |

Table v. Definitions of the neurological condition categories used in the analysis.

**Supplementary Results**

| Year | All papers | Neurology (%) |
| --- | --- | --- |
| 1991 | 21 | 1 (4.8) |
| 1992 | 11 |  |
| 1993 | 29 |  |
| 1994 | 37 |  |
| 1995 | 58 |  |
| 1996 | 57 |  |
| 1997 | 80 |  |
| 1998 | 92 | 1 (1·1) |
| 1999 | 107 | 1 (0·9) |
| 2000 | 104 | 2 (1·9) |
| 2001 | 126 | 3 (2·4) |
| 2002 | 142 | 4 (2·8) |
| 2003 | 193 | 3 (1·6) |
| 2004 | 244 | 8 (3·3) |
| 2005 | 341 | 5 (1·5) |
| 2006 | 429 | 12 (2·8) |
| 2007 | 462 | 7 (1·5) |
| 2008 | 496 | 13 (2·6) |
| 2009 | 882 | 27 (3·1) |
| 2010 | 1703 | 53 (3·1) |
| 2011 | 1971 | 53 (2·7) |
| 2012 | 2355 | 78 (3·3) |
| 2013 | 2994 | 121(4·0) |
| 2014 | 3374 | 153 (4·5) |
| 2015 | 3798 | 209 (5·5) |
| 2016 | 3918 | 286 (7·3) |
| 2017 | 4303 | 350 (8·1) |
| 2018 | 4973 | 401 (8·1) |

Table vi. Numbers of papers relating to the use of EHRs and Routinely collected data retrieved each year in PubMed search.

|  | Multiple Sclerosis (n=139, %) | Epilepsy/ Seizure  (n=63, %) | Parkinson’s Disease (n=29, %) | Migraine (n=16, %) | Other Headache (n=14, %) | Multiple Conditions (n=12, %) | Neurodegenerative Disorders  (n=8, %) | Neuromuscular Disorders (n=9, %) | Other (n=97, %) |
| --- | --- | --- | --- | --- | --- | --- | --- | --- | --- |
| Hospital | 31 (22·3) | 23 (36·5) | 14 (48·3) | 1 (6·3) | 7 (50·0) | 8 (66·7) | 4 (50·0) | 4 (44·4) | 65 (67·0) |
| Claims | 49 (35·3) | 4 (6·3) | 4 (13·8) | 3 (18·8) |  | 1 (8·3) | 1 (12·5) | 1 (11·1) | 3 (3·1) |
| Clinic | 21 (15·1) | 12 (19·0) | 5 (17·2) | 8 (50·0) | 5 (35·7) | 1 (8·3) | 1 (12·5) | 3 (33·3) | 2 (2·1) |
| Multicentre | 21 (15·1) | 10 (15·9) | 1 (3·4) | 3 (18·8) | 1 (7·1) |  | 1 (12·5) | 1 (11·1) | 6 (6·2) |
| Veterans | 3 (2·2) | 2 (3·2) | 3 (10·3) | 1 (6·3) | 1 (7·1) |  |  |  | 14 (14·4) |
| Primary Care | 8 (5·8) | 5 (7·9) | 2 (6·9) |  |  | 1 (8·3) | 1 (12·5) |  | 2 (2·1) |
| Pharmaceutical | 3 (2·2) | 3 (4·8) |  |  |  |  |  |  | 2 (2·1) |
| Other | 3 (2·2) | 4 (6·3) |  |  |  |  |  |  | 3 (3·1) |

Table vii. Primary condition focus split by data type used.

|  | Hospital (n=157, %) | Claims (n=66, %) | Clinic Data (n=58, %) | Multi-centre (n=44, %) | Veterans and military (n=24, %) | Primary Care (n=18, %) | Pharmaceutical (n=9, %) | Other (n=10, %) |
| --- | --- | --- | --- | --- | --- | --- | --- | --- |
| Descriptive | 107 (68·2) | 38 (57·6) | 42 (72·4) | 21 (47·7) | 17 (70·8) | 7 (38·9) | 6 (66·7) | 5 (50·0) |
| Regression | 25 (15·9) | 19 (28·8) | 8 (13·8) | 12 (27·3) | 1 (4·2) | 2 (11·1) |  | 1 (10·0) |
| Survival Analysis | 8 (5·1) | 2 (3·0) | 4 (6·9) | 3 (6·8) |  | 1 (5·5) | 2 (22·2) |  |
| Administrative Algorithm | 2 (1·3) | 3 (4·6) |  | 3 (6·8) | 1 (4·2) | 6 (33·3) |  |  |
| Machine Learning | 3 (1·9) |  | 1 (1·7) | 1 (2·3) |  |  |  | 2 (20·0) |
| NLP | 4 (2·5) | 1 (1·5) | 1 (1·7) |  | 4 (16·6) |  |  |  |
| Propensity Scoring | 2 (1·3) | 2 (3·0) | 1 (1·7) | 1 (2·3) |  |  |  | 2 (20·0) |
| ANOVA | 2 (1·3) | 1 (1·5) |  |  |  | 1 (5·5) |  |  |
| Other | 4 (2·5) |  | 1 (1·7) | 3 (6·8) | 1 (4·2) | 1 (5·5) | 1 (11·1) |  |

Table viii. Data Type split by statistical analysis used.
